# Endocervical gastric‐type AIS: a rare histologic finding that further supports the hypothesis of a pyloric‐type metaplastic origin

**DOI:** 10.1111/his.70150

**Published:** 2026-04-01

**Authors:** Michele Paudice, Veronica Parrella, Giulia Scaglione

**Affiliations:** ^1^ Department of Integrated Diagnostic and Surgical Sciences (DISC) University of Genoa Genoa Italy; ^2^ IRCCS Azienda Ospedaliera Metropolitana (AOM) Genoa Italy; ^3^ Pathology Unit ASL2 Liguria Savona Italy; ^4^ Pathology Unit, Department of Woman and Child's Health and Public Health Sciences Fondazione Policlinico Universitario Agostino Gemelli IRCCS Rome Italy

## Abstract

**Background:**

Gastric‐type adenocarcinoma in situ (gAIS) is a rare, HPV‐independent precursor of cervical adenocarcinoma with incompletely defined histogenesis.

**Aims:**

To report a case of gAIS and its association with pyloric‐type gastric metaplasia.

**Materials and Methods:**

A case of gAIS in a 70‐year‐old woman with atypical glandular cells (Pap test) and negative HPV molecular testing was evaluated histologically and immunohistochemically.

**Results:**

Conization revealed atypical glandular epithelium. The lesion was CK7/CDX2 positive, with mutant‐type p53 expression, and negative for CK20, PAX8 and p16. Adjacent pyloric‐type gastric metaplasia was identified.

**Discussion:**

The association with pyloric‐type metaplasia supports a possible metaplastic origin and highlights diagnostic challenges in limited samples.

**Conclusion:**

This case supports a possible link between gAIS and pyloric‐type gastric metaplasia, underscoring the importance of accurate recognition in HPV‐independent cervical lesions.

**Abbreviations**
aLEGH: atypical lobular endocervical glandular hyperplasia
gAIS: gastric‐type adenocarcinoma in situ
HPV: human papillomavirus

The identification of human papillomavirus (HPV)–independent precursors of cervical adenocarcinoma has become increasingly important in the era of HPV‐based screening. In their work, Talia *et al*. defined the morphologic and immunophenotypic characteristics of gastric‐type adenocarcinoma in situ (gAIS) of the cervix.^1^ Their work clarified the features of this entity, including gastric‐type mucinous differentiation with preserved glandular architecture, mild atypia and a characteristic immunoprofile of CK7 and MUC6 positivity, variable CDX2/CK20/PAX8 expression, negative or mosaic p16, variable p53 immunostaining (including both wild‐type and mutation‐type patterns) and HPV negativity.

We wish to contribute to this field by presenting a case of gAIS in a 70‐year‐old South American woman whose Pap test revealed atypical glandular cells with concurrent negative HPV testing. Cervical conization showed isolated foci of atypical glandular epithelium involving markedly ectatic endocervical crypts. These areas demonstrated an abrupt transition from normal mucosa, nuclear pseudostratification, low‐grade hyperchromasia and scattered apical mitoses, without evident apoptotic bodies or goblet cell differentiation; these features were partially reminiscent of HPV‐related epithelial changes. The lesion was completely excised, and margins were negative.

Immunohistochemistry revealed positivity for CK7 (focal) and CDX2 (diffuse), with aberrant (mutation‐type, overexpression) p53 staining and negativity for CK20, PAX8 and p16. Some crypts in proximity displayed a monolayer of pyloric‐type gastric metaplasia (Figure 1). Importantly, the atypical foci were restricted to dilated crypts without evidence of atypical lobular endocervical glandular hyperplasia (aLEGH). Only small foci of LEGH without atypia were identified at a distance from the gAIS. This finding supports the hypothesis – also suggested by Talia *et al*. – that gAIS may arise directly from pyloric‐type gastric metaplasia rather than exclusively through progression from LEGH.^1^

**Figure 1:**
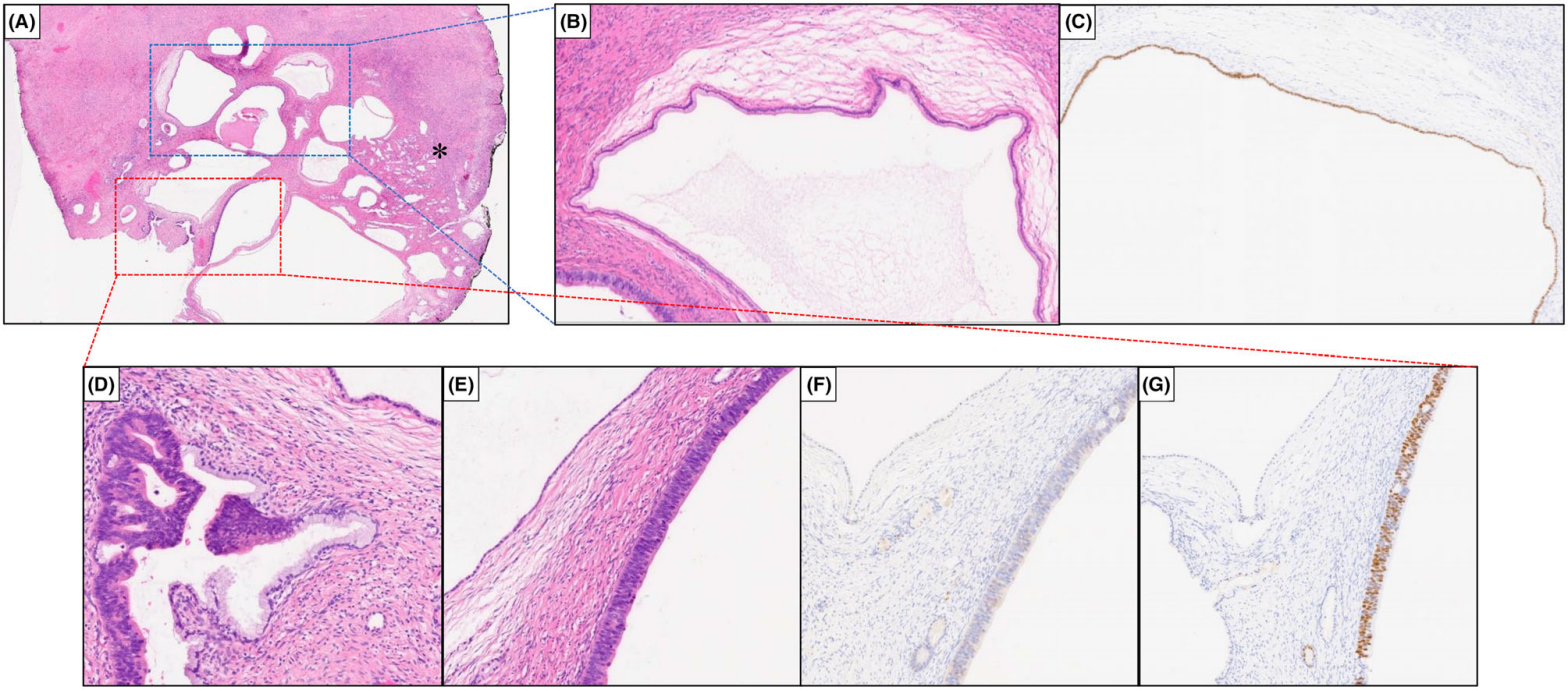
Histologic features of the cervical cone specimen. **(A)** Low‐power view of the cervical cone showing epithelial remodelling with areas of glandular ectasia and focal lobular endocervical glandular hyperplasia (LEGH) (*) with preserved stromal architecture (EE 2×). **(B)** Focus of pyloric mucinous metaplasia characterized by columnar cells with apical intracytoplasmic mucin and **(C)** p53 aberrant (IHC 10×). **(D, E)** Area of in situ adenocarcinoma exhibiting moderate atypia, with an altered nuclear‐cytoplasmic ratio, hyperchromasia and architectural irregularity with loss of cell polarization and focally with cribriform appearance (EE 20×). **(F)** Immunohistochemistry for p16 demonstrated negative staining, consistent with the absence of a block‐type pattern (IHC 20x); Immunohistochemistry for p53 **(G)** showed expression in more than 70% of neoplastic cells, with strong nuclear staining (IHC 20×).

This case highlights the diagnostic challenges associated with gAIS, which may mimic usual‐type adenocarcinoma in situ, particularly in small or fragmented samples. In the absence of clear HPV‐related features or diffuse block‐type p16 staining, an expanded immunohistochemical panel is essential.^2^, ^3^

This case contributes additional observations to the expanding spectrum of gAIS. The close proximity of benign pyloric‐type mucosa to gAIS raises the possibility that gastric‐type metaplasia may act as a precursor lesion. Furthermore, the morphological features observed in this case suggest that exclusive reliance on overt gastric differentiation may not capture the full histologic range of this entity. Indeed, the partial morphologic overlap with usual‐type adenocarcinoma in situ and reactive glandular changes highlights the diagnostic challenges posed by gAIS, particularly in the absence of an appropriate immunohistochemical panel. Had conization not been performed, a true precursor of gastric‐type adenocarcinoma – known for its aggressive behaviour and poorer prognosis compared with HPV‐associated adenocarcinomas – might have been missed.^1^ Given the aggressive behaviour of its invasive counterpart, accurate recognition of gAIS is clinically significant and may necessitate a more vigilant management approach than that employed for HPV‐associated AIS.^4^, ^5^ This underscores the need for heightened clinical suspicion and careful colposcopic and histologic evaluation in HPV‐negative patients presenting with cytological glandular atypia.

Increased awareness of the morphologic spectrum and precursor pathways of gastric‐type lesions is therefore critical for accurate diagnosis and optimal patient management and continued reporting of gAIS – particularly cases arising in the absence of aLEGH – may further refine diagnostic criteria and deepen our understanding of HPV‐independent carcinogenesis of the cervix.

## Author contributions

All listed authors contributed to the production of this manuscript and are listed in the appropriate order. All authors gave their approval for the publication of the final version of the manuscript.

## Funding information

This research received no external funding.

## Conflicts of interest

The authors declare no conflict of interest.

## Data Availability

The data that support the findings of this study are available from the corresponding author upon reasonable request.
